# The evolving prehospital care: a 12-year retrospective analysis

**DOI:** 10.1186/s13049-026-01651-z

**Published:** 2026-07-01

**Authors:** Helena Johansson, Daniel Wilhelms, Jens Wretborn

**Affiliations:** 1 Department of Biomedical and Clinical Sciences, Linköping University, Linköping, Sweden; 2 Clinical Department of Emergency Medicine in Linköping, Region Östergötland, Linköping, Sweden; 3 Falck Emergency Östergötland, Linköping, Sweden

**Keywords:** Acute care chain, Emergency medical services, Patient population, Temporal trends, Triage acuity, Mortality, Admission, Emergency department

## Abstract

**Background:**

Emergency medical services (EMS) are one of the main interfaces between acutely ill patients in the prehospital environment and hospital-based care. Yet longitudinal, population-level studies of EMS patient populations remain limited. We aimed to characterize temporal trends in EMS demand, triage acuity, hospital admission, and short-term mortality among patients managed by EMS and transported to emergency departments (EDs) in a Swedish healthcare system from 2011 to 2022.

**Methods:**

This was a retrospective, population-based study of all primary EMS assignments in Region Östergötland, Sweden (catchment population 456,000), from 2011 to 2022. Data were extracted from linked electronic health records encompassing EMS, ED, and inpatient care. Triage acuity was classified using the Rapid Emergency Triage and Treatment System (RETTS). The primary outcome was the temporal trend in triage acuity among EMS patients. Secondary outcomes included hospital admission rates, 1, 7, 30, and 90-day mortality, and non-transport disposition rates. Proportional-odds ordinal regression and multivariable logistic regression were used to assess temporal trends. Descriptive statistics were used to summarize demographic data.

**Results:**

Of 474,647 included primary assignments, 394,580 (83.1%) resulted in ED transport. Triage acuity shifted progressively toward higher-severity categories. The combined proportion of acuity levels 1 and 2 (highest) increased from 32% to 57%, with an 8% annual increase in odds of higher classification (OR 1.08 per year, 95% CI 1.08–1.09). Non-transport dispositions rose from 5% to 25% of primary assignments. The hospital admission rate among EMS-transported patients was constant at approximately 50%, but their share of all ED-originating hospital admissions increased from 42% to 57%. Short-term mortality was stable; triage acuity was the strongest predictor of death at all time points (OR 1.14–5.15 per level).

**Conclusions:**

Over 12 years, EMS-transported patients presented with progressively higher triage acuity while constituting an increasing share of hospital admissions. These trends, concurrent with a fivefold expansion in non-transport dispositions, reflect a shift in the role of EMS toward field-based clinical assessment and disposition. These findings have implications for EMS resource planning, triage system evaluation, and the integration of prehospital data into hospital capacity management.

**Supplementary Information:**

The online version contains supplementary material available at 10.1186/s13049-026-01651-z.

## Background

Emergency Medical Services (EMS) form an essential link between acutely ill or injured patients in the prehospital environment and Emergency Departments (EDs). Over the past three decades, major shifts have taken place both in the organization of EMSs and EDs, as well as the underlying patient demographics [1–3]. Specifically, an aging population has increased the burden of multimorbidity and acute-on-chronic illness. Improved prevention and pharmacotherapy for cardiovascular and respiratory diseases have reduced fatality rates from these conditions, but increased the proportion of older adults with complex care needs [3–5]. Also, substantial advances in road and vehicle safety, as well as an overall higher risk-awareness within the community [6] have led to decreased high-energy trauma patient volumes in both EMS and ED. These demographic and societal changes have reshaped both the spectrum of EMS encounters as well as the acuity of patients transported to the ED [1, 5, 6].

Research in prehospital emergency medicine has primarily focused on specific patient groups, including out of hospital cardiac arrest (OHCA), major trauma, acute coronary syndromes or stroke [7–13] and seen major advances in prehospital resuscitation and critical care management. However, these patients constitute a fraction of the encounters managed by EMS systems and few investigations have examined the entire population of patients encountered by EMS and their outcomes. Understanding how EMS demand and utilization in terms of case acuity, transports, admissions and mortality have evolved is essential for strategy planning of staff and resources, as well as the development of clinical EMS practices.

In Sweden, healthcare is tax-funded, with a small symbolic fee for emergency care, and delivered through 21 regional healthcare systems. The EMS is staffed with two personnel where at least one needs to be a registered nurse or specialist nurse (specialised in prehospital emergency care, emergency care, anesthesiological care or intensive care). Since the early 2000s, Swedish prehospital care has undergone professionalization, widespread adaptation to electronic health records (EHR) and systematic adoption of triage systems, of which Rapid Emergency Triage and Treatment System (RETTS) is the most widespread in both EMS and EDs. The EHR adoption together with the comprehensive healthcare system makes linked healthcare records between pre-hospital and hospital care possible and enables longitudinal investigation of the emergency care chain.

### Aim

The aim of this study was to describe the temporal trends in EMS demand, case-mix, triage acuity, hospital admission, and short-term mortality among EMS-transported ED patients in a Swedish healthcare system, and to describe concurrent changes in non-transport dispositions and the distribution of transports across EDs.

## Methods

This was a retrospective observational study of all primary EMS assignments in Region Östergötland, Sweden, from January 1st 2011, through December 31st 2022. Between these years the population in the region increased from 430,000 to 472,000 and became older, with the proportion of residents aged 65 years and older increasing from 87,236 in 2011 to 101,247 in 2022 [14]. The regional healthcare system is served by two EMS subcontractors and three EDs; one university hospital, one urban community hospital, and one rural community hospital. EMS dispatch is managed by a national dispatch service co-owned and used by most healthcare systems in Sweden, which receives calls to the national emergency number 112. The dispatch service categorizes assignments according to the national standard for ambulance data, adapted from the National Emergency Medical Information System (NEMSIS) [15]. One of the three categories is primary assignment, which definition is an “Assignment to the scene of illness, injury, or incident” [15]. In a primary assignment, the EMS crew makes individual assessments of the patients acuity level, medical and care needs and based on this assessment the patients are either transported to an ED or not transported by EMS, which could mean they are referred to another transportation mode to ED, referred to primary care, or referred to self-care.

Ethical approval for this study was obtained from the Swedish Ethical Review Authority (permit number 2022-03404-01).

### Data sources and measurements

We included all primary EMS-assignments, where a patient had been assessed by an EMS nurse. Secondary transport assignments (inter-facility transportation), patients declared deceased at the scene, and patients transported to EDs outside the region were excluded.

Patient acuity was classified by EMS personnel using RETTS, which integrates an assessment of the chief complaint (Emergency Signs and Symptoms) with vital sign data to assign an acuity, ranging from 1 (red, highest acuity) through 4 (green, lowest acuity). RETTS has been used since 2010, is updated annually, and underwent a major revision in 2014 which was aimed to enhance standardization and give a clearer structure. The revision primarily clarified the description of acuity levels 1 and 2 in the chief complaint assessments in specific ESS categories with limited or no impact on the overall triage process and assessment. Electronic health records (EHRs) encompassing EMS, ED and inpatient data were fully implemented by the start of the study period.

### Variables

The primary outcome was the temporal trend in prehospital triage acuity among EMS patients transported to the ED over the study period. Secondary outcomes were dispersion of EMS patients between the three EDs; the proportion of EMS-transported patients subsequently admitted to hospital; 1, 7, 30, and 90 day all-cause mortality following EMS assignment in transported patients; the proportion of primary assignments resulting in non-transport (on-scene treatment, referral or other disposition). We also collected the annual number of ED visits admitted to hospital to calculate the proportion of patients admitted to hospital that were transported by EMS.

### Statistical analysis

Descriptive statistics are reported as proportions and means with standard deviations. For the primary outcome, a cumulative proportional-odds ordinal regression model was used to assess the association between calendar year and triage acuity level, adjusting for age, sex, and their two-way interactions. The two-way interactions were assessed to be able to further interpret the results of the regression model. The ordered triage variable was inverted (1 being least acute) so that increasing values reflected increasing acuity to facilitate interpretation of odds ratios (ORs). Positive log-odds coefficients indicate increased probability of assignment to higher-acuity categories. Model fit was assessed by likelihood ratio test against an intercept-only model.

For secondary outcomes, the proportions of EMS-transported patients admitted to hospital was calculated annually and expressed both as a proportion of all EMS-transported patients and as a proportion of all ED admissions. Binomial logistic regression models estimated ORs for 1, 7, 30 and 90 day all-cause mortality, adjusting for age, sex, acuity level and calendar year. The proportion of non-transport dispositions was calculated annually as a share of all primary assignments.

Effect estimates were reported as ORs with 95% confidence intervals (CIs). A two-sided p-value < 0.05 was considered statistically significant. Model performance was primarily assessed based on the magnitude and precision of the estimated associations, as reflected by ORs and corresponding 95% CIs.

Missing triage data were handled by only analysing complete cases in the triage and mortality models. Otherwise, no data were missing for age, sex, calendar year or mortality outcomes. All analyses were performed using R (version 4.4.0) and ggplot2 package (version 3.4.1).

### Bias

Adjustments were made in the logistic regression models for age, sex, acuity level and calendar year, to be able to see each variable’s actual development over the time period and minimize the risk of bias when interpreting the results.

### Study size

No formal sample size calculation was done as this was a whole-population study.

## Results

**Fig. 1:**
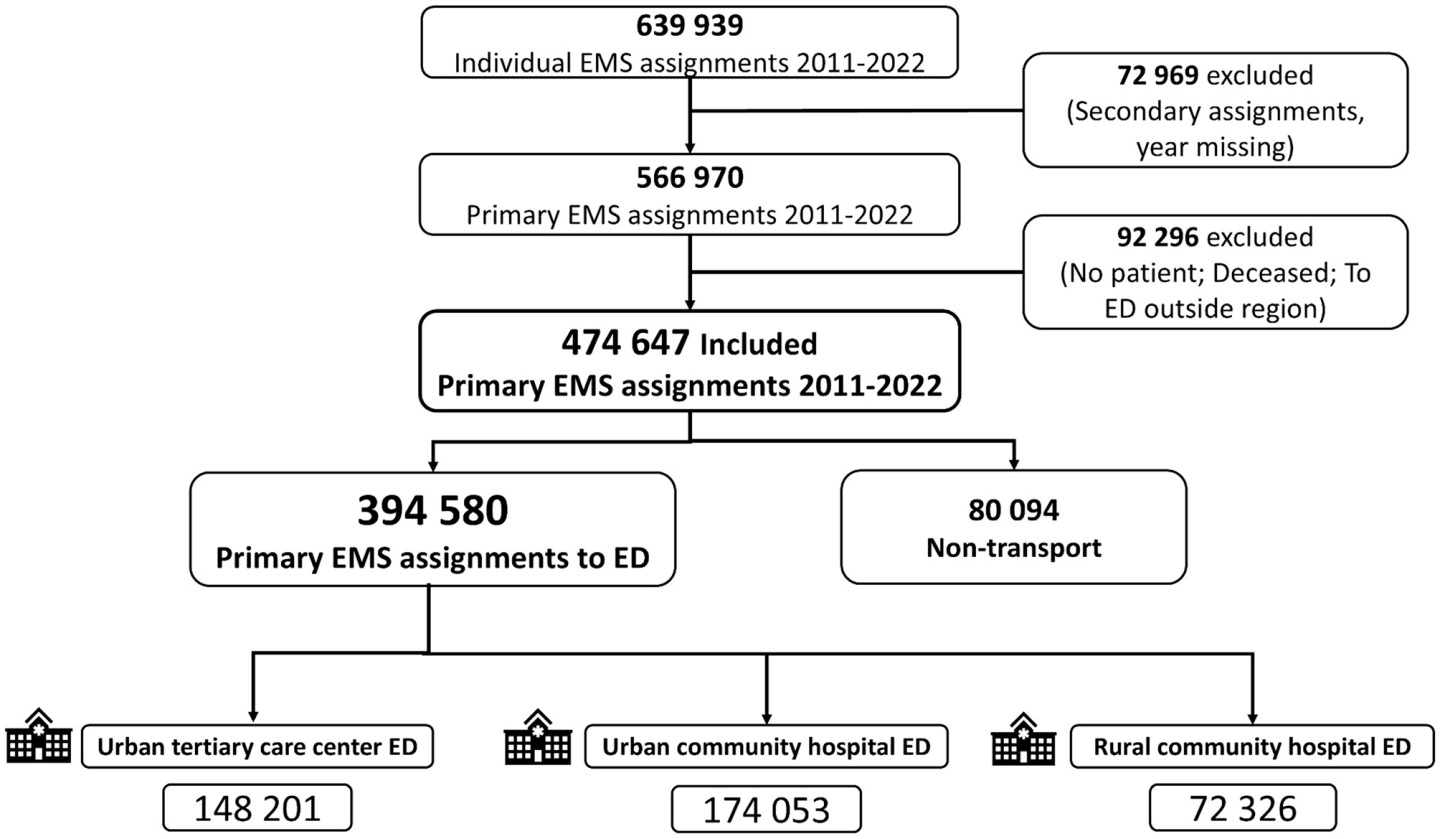
Flowchart of included patients

**Fig. 2:**
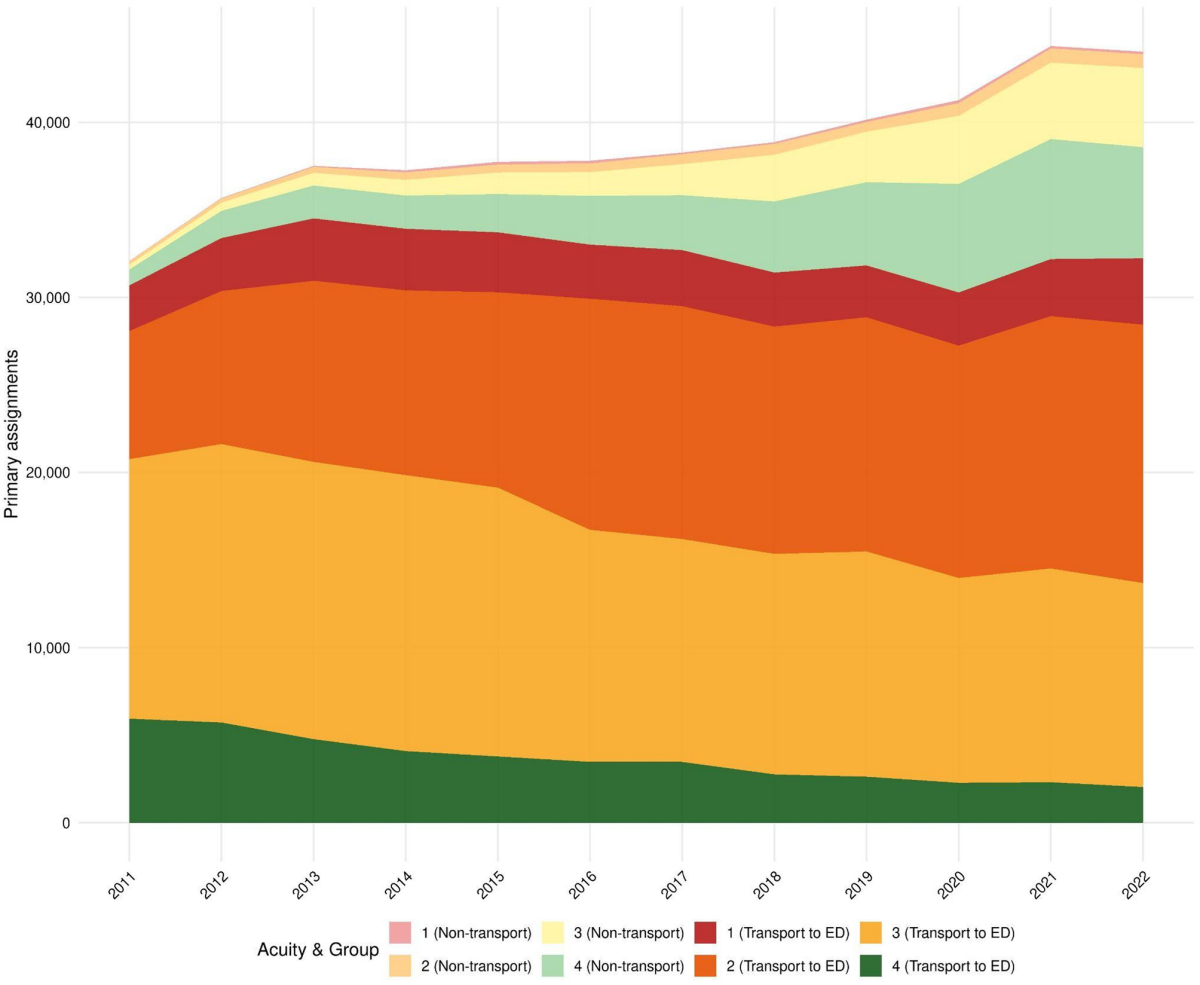
Number of primary assignments assessed by EMS where patients was either transported to an emergency department or referred by EMS, divided by triage acuity

During the 12-year study period, 566,970 EMS assignments were registered, of which 474,647 were primary assignments meeting inclusion criteria (Fig. 1). Of these, 394,580 (83%) resulted in patient transport to one of the three regional EDs and constituted the study cohort. The remaining 80,094 (17%) were assignments in which patients were referred on scene to another level of care or mode of transportation. Annual ED transport volume did not show a linear trend over the study period, varying between 30,604 (2020) to 34,733 (2013). Non-transport assignments increased from 2,219 (5% of primary assignments) in 2011 to 12,351 (25%) in 2022 (Fig. 2).

### Triage acuity

A progressive shift toward higher acuity was observed over the study period among transported patients (Table 1; Fig. 2). The proportion of patients assigned acuity level 4 decreased from 5995 (19%) in 2011 to 2058 (6%) in 2022, while acuity level 2 increased from 7330 (24%) to 14,791 (46%) and acuity level 1 rose from 2613 (8%) to 3812 (12%). Missing triage data were minimal (1,536 of 394,580 assignments; 0.4%). For non-transported patients, distribution of acuity levels for large unchanged during the study period with predominantly acuity levels 3 and 4 (Fig. 2). Calendar year (OR 1.07), older age (OR 1.16), as well as male gender (OR 1.17) was associated with higher triage acuity in all primary assignments between 2011 and 2022.

### EMS patients transported to ED

Patients transported by EMS to an ED were 52% female throughout the study period. Mean age increased from 64.1 years (SD 22.7) in 2011 to 66.2 years (SD 22.9) in 2022 (Table 1, Additional file 1). The distribution of transports by time of day was consistent across all years, with approximately 57% occurring during daytime, 22% during evening, and 21% during nighttime hours (Table 1, Additional file 1).

**Table 1:** Descriptive characteristics of all primary EMS assignments resulting in ED transport 2011 to 2022. Data are shown for the entire study period (mean and proportion or SD for each variable), as well as separately for 2011 and 2022 (total n and proportion or SD for each variable)

Primary assignments to ED	2011 to 2022 (Mean)	2011	2022
**N**	32881.7	31,043	32,440
Women (n (%))	17029.3 (51.8)	16,085 (51.8)	16,707 (51.5)
Age (mean (SD))	63.1 (24.4)	64.1 (22.7)	66.2 (22.9)
**Time of Day**			
Day (06–17) (n (%))	18629.4 (56.7)	17,345 (55.9)	18,376 (56.6)
Evening (17–22) (n (%))	7404.3 (22.5)	7096 (22.8)	7394 (22.8)
Night (22 − 06) (n (%))	6848.0 (20.8)	6602 (21.3)	6670 (20.6)
**Triage Acuity**			
Acuity 1 (Red) (n (%))	3239.6 (9.9)	2613 (8.4)	3812 (11.8)
Acuity 2 (Orange) (n (%))	12042.7 (36.6)	7330 (23.6)	14,791 (45.6)
Acuity 3 (Yellow) (n (%))	13824.3 (42.0)	14,855 (47.9)	11,693 (36.0)
Acuity 4 (Green) (n (%))	3647.1 (11.1)	5995 (19.3)	2058 (6.3)
Missing acuity (n (%))	128.0 (0.4)	250 (0.8)	86 (0.3)
**Distribution across EDs**			
ED Urban tertiary care center (n (%))	12350.1 (37.6)	11,643 (37.5)	11,669 (36.0)
ED Urban community hospital (n (%))	14504.4 (44.1)	13,545 (43.6)	15,131 (46.6)
ED Rural community hospital (n (%))	6027.2 (18.3)	5855 (18.9)	5640 (17.4)

In the proportional-odds ordinal regression model (*n* = 393,044), calendar year was the strongest predictor of higher acuity (OR 1.08 per year, 95% CI 1.08–1.09, *p* < 0.001), corresponding to an 8% annual increase in odds of assignment to a higher-acuity category (Table 2). Age had a small positive effect (OR 1.003 per year of age, 95% CI 1.00–1.00, *p* < 0.001). Female sex was associated with lower odds of higher acuity level (OR 0.84, 95% CI 0.84–0.86, *p* = 0.066). The interaction between sex and age was statistically significant (OR 0.99, 95% CI 0.99–1.00, *p* = 0.002), indicating an attenuated age effect among women.

**Table 2:** Multivariable cumulative odds ratios for EMS triage severity (*n* = 393,044)

Predictor	OR	95% CI	*p*-value
Sex (Female vs. Male)	0.84	0.84–0.86	0.066
Age (Per year)	1.00	1.00–1.00	< 0.001
Calendar Year (per year)	1.08	1.08–1.09	< 0.001
Sex * Age	0.99	0.99–1.00	0.002

Model: χ^2^ (7) = 10.020, *p* < 0.001

**Fig. 3:**
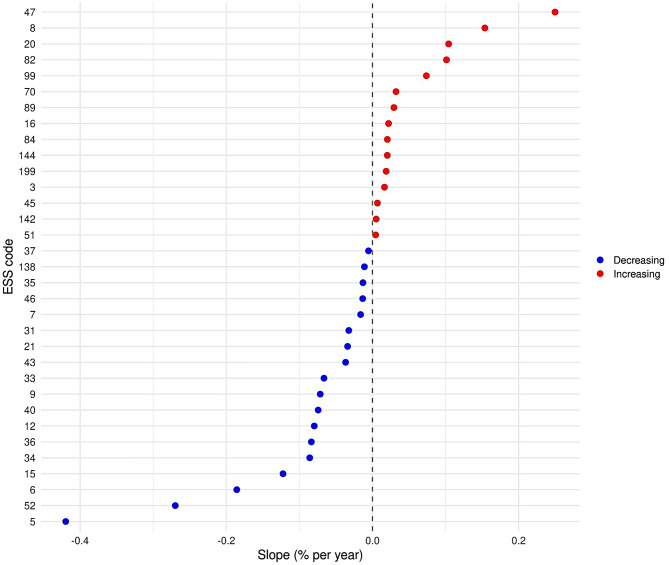
The top 33 changing ESS-codes from 2011 to 2022, 18 decreasing (blue) and 15 increasing (red)

Figure 3 shows the 33 ESS-codes that changed the most during the study period, 18 decreasing and 15 increasing in use (Fig. 3). An explanation of all ESS-codes in Fig. 3 are available in Table 3 below.

**Table 3:** ESS-codes represented in Fig. 3, freely translated to english

ESS-codes		
3 Coughing up blood/ Epistaxis/Tonsil bleeding	31 Chest or thoracic trauma	51 Adrenal insufficiency/ Addison’s disease
5 Chest pain/Chest discomfort	33 Hand/arm injury	52 Unspecified psychiatric problem
6 Abdominal pain/Flank pain	34 Hip/thigh/knee/lower leg/foot injury	70 Lab results triage algorithm
7 Vomiting blood/Blood in stool/Rectal symptoms/ Gastrointestinal bleeding	35 Burn/inhalation/electrical/ lightning/cold injury	82 Anxiety
8 Nausea/Vomiting/Diarrhea	36 Drowning/Wound	84 Psychotic symptoms
9 Seizures	37 Eye injury/eye problem	89 Dementia/Disorientation/ Organic psychiatric disorder
12 Stroke/TIA	40 Intoxication/Poisoning	99 Suicide risk assessment
15 Pain in extremity/Swelling in extremity/Leg edema	43 Allergy/Medication reaction/Rash	138 Trauma alarm algorithm (pediatric version)
16 Urinary retention/Hematuria/ Urinary problems	45 ENT problem	142 Physical abuse (pediatric version)
20 Fainting/Collapse/Loss of consciousness	46 Foreign body in nose/airway/esophagus	144 Sore throat/cough/cold/ mouth blisters (pediatric version)
21 Female genital complaints/ Vaginal bleeding	47 Infection/Fever/Local infection/Sepsis	199 Suicide risk assessment (pediatric version)

### Outcomes of patients transported to an ED

Among EMS-transported patients, the annual hospital admission rate remained consistent between 46% and 54% throughout the study period (Table 4, Additional files 1). Notably, the number of patients (all patients in the ED, both EMS and “walk-ins”) admitted to hospital from the EDs decreased from 35,490 in 2011 to 31,062 in 2022. Consequently, EMS transported patients accounted for a steadily increasing share of all hospital admissions, rising from 42% in 2011 to 57% in 2022.

Crude mortality rates were consistent across the study period for all four time periods (1-, 7-, 30-, and 90-days), with the exception of small peaks in 2020 and 2021 (Table 4, Additional files 1). 90-day mortality ranged from 3.4% to 3.8% annually.

**Table 4:** Hospital admissions and mortality among patients transported by EMS to an ED 2011 to 2022. Data are shown for the entire study period (mean and proportion or SD for each variable), as well as separately for 2011 and 2022 (total n and proportion or SD for each variable)

	2011 to 2022 Mean	2011	2022
Admissions (all EDs) (*n*(%))	16346.6 (49.7)	14,360 (46.3)	17,606 (54.3)
Mortality within 1 day (n(%))	348.2 (1.1)	304 (1.0)	369 (1.1)
Mortality within 7 days (n(%))	472.7 (1.4)	393 (1.3)	468 (1.4)
Mortality within 30 days (n(%))	862.5 (2.6)	732 (2.4)	920 (2.8)
Mortality within 90 days (n(%))	1119.8 (3.4)	1007 (3.2)	1234 (3.8)

In the adjusted model, acuity level was the strongest predictor of mortality at all time points (OR 1.14–5.15 per higher acuity level; Table 5). The association was most pronounced for 1-day mortality (OR 5.15, *p* < 0.05). Increasing age was associated with higher mortality across all time horizons (OR 1.04–1.07 per year of age). Female sex was protective at all time points, with the greatest effect at 30 days (OR 0.71, *p* < 0.05). Calendar year showed a marginal negative association with 7-days mortality (OR 0.99), *p* < 0.05) (Table 5).

**Table 5:** Predictors of mortality following EMS assignment (*n* = 393,044). (Complete-case analysis (*n* = 393,044 of 394,580; 1,536 missing Triage acuity, 0.39%))

Predictor	1 day mortality OR(95% CI)	7 days mortality OR(95% CI)	30 days mortality OR(95% CI)	90 days mortality OR(95% CI)
Age (per year)	1.04(1.04–1.04)*	1.07(1.06–1.07)*	1.07(1.06–1.07)*	1.06(1.06–1.06)*
Gender (F vs. M)	0.81(0.75–0.87)*	0.75(0.71–0.79)*	0.71(0.68–0.74)*	0.74(0.71–0.77)*
Triage (per level)	5.15(4.90–5.43)*	2.26(2.18–2.34)*	1.37(1.34–1.41)*	1.14(1.12–1.17)*
Year (per year)	1.00(0.99–1.01)	0.99(0.98-1.00)*	1.00(1.00-1.01)	1.00(1.00-1.01)

*p < 0.05

### Distribution of transports across EDs

**Fig. 4:**
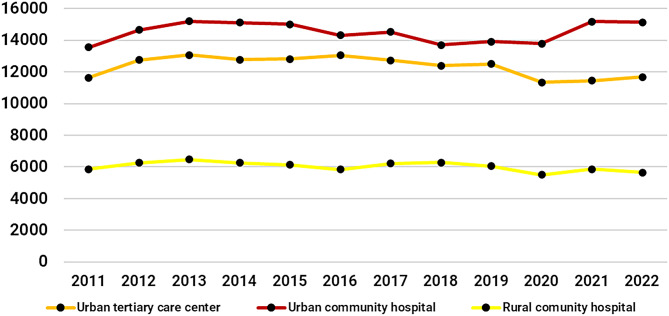
Primary EMS assignments to each ED between 2011 and 2022

Patients were most frequently transported to the urban community hospital ED (14504.4, 44.1%), followed by the university hospital ED (12350.1, 37.6%), and the rural community hospital ED (6027.2, 18.3%). The distribution shifted over time: the urban hospital ED share increased from 43.6% to 46.6% (Table 1; Fig. 4). From the year 2020 a larger increase in patients transported by EMS to ED was seen at the urban community hospital in comparison to the other two EDs (Fig. 4).

## Discussion

In this population-based study of nearly 400,000 primary EMS assignments resulting in ED transport over a 12-year period, we observed three principal findings. First, prehospital acuity level among EMS-transported patients shifted progressively toward higher-severity categories, with an estimated 8% annual increase in the odds of assignment to a higher triage level. Second, while the proportion of EMS-transported patients admitted to hospital remained consistent at approximately 50%, the overall rate of hospital admission from the ED declined, such that EMS-transported patients accounted for a growing share of all hospital admissions. Third, short-term mortality remained largely unchanged over time despite an aging study population, and prehospital triage acuity was the strongest predictor of death at all time horizons examined.

The progressive increase in acuity has several possible explanations. Population aging, as reflected in a two-year increase in mean patient age over the study period, may have contributed, as older patients tend to present with more acute and complex conditions [16]. However, the effect of calendar year on triage acuity persisted after adjustment for age, suggesting that demographic shifts alone do not account for the observed trend. The 2014 RETTS revision may have caused limited reclassification across assessment categories and acuity levels, but it is unlikely to explain more than a small fraction of the observed temporal increase in acuity. A change of chief complaint coding, and thereby the downstream acuity by the triage algorithm is unlikely based on the subtle changes in ESS-coding trends seen during the study period. Suggesting that the observed acuity trend may represent changes in disease and presentation severity.

A fivefold increase in non-transport dispositions, from 5% to 25% of primary assignments, implies that lower-acuity patients were increasingly managed on scene and referred to alternative care pathways, thereby concentrating higher-acuity patients among those transported to the ED. This is consistent with the broader transformation of Swedish EMS from a transport-centric service toward a system performing clinical assessment and disposition in the field [17]. Similar trends in non-transport rates have been reported in other Swedish healthcare systems and internationally, raising questions about patient safety in the non-transported population that merit further investigation [18, 19].

Mortality was mostly unchanged across the study period, with modest increases in 30- and 90-day mortality that were of borderline clinical significance. Peaks in 2020 and 2021 likely reflect the impact of the COVID-19 pandemic. In multivariable models, triage acuity demonstrated a strong and graded association with mortality at all time points, with odds ratios ranging from 1.09 to 1.48 per acuity level.

Sweden has experienced the largest reduction in hospital beds per capita of any European country over the past two decades, resulting in higher bed-occupancy rates and increased ED crowding [20]. The finding that EMS-transported patients constituted an increasing proportion of all hospital admissions from the ED, rising from 42% to 57%, warrants attention from a health-system perspective. It occurred not because EMS patients were admitted more frequently but because overall ED admission rates declined. A possible reason for this is the aging population, since previous studies in the ED-setting have shown higher admission rates among the older/frail patients [21]. Whether the declining overall admission rate reflects improved outpatient alternatives, more restrictive admission thresholds driven by bed constraints, or changes in the non-EMS ED population cannot be determined from our data. Regardless of the mechanism, the growing dominance of EMS-transported patients among hospital admissions underscores their importance as a marker of acute inpatient demand.

The number of patients transported to ED by EMS was at its lowest number in the year of 2020, probably reflecting effects of the covid-19 pandemic. The following years the number of EMS transported patients to ED regained to previous levels, although a shift in distribution between the EDs could be seen. The urban community hospital had a larger increase in EMS transported patients compared to the other two EDs, possibly reflecting socioeconomic differences, which have been previously described in the region [22]. Whether the Covid-19 pandemic further reinforced these differences and their impact on EMS utilization should be further investigated.

This study encompasses the entire EMS-to-ED population of a defined geographic region over 12 years, avoiding the selection bias inherent in studies restricted to patient subsets such as specific diagnoses or triage categories. Data completeness was high, with triage data missing in only 0.39% of assignments and vital-sign documentation available for 98.3% of transports, reflecting robust electronic health record implementation. Several limitations should be acknowledged. The study is based on a single Swedish region, and generalizability to other healthcare systems may be limited, although the demographic composition of this region is relatively comparable to large parts of Scandinavia and Northern Europe. The retrospective design precludes causal inference; observed trends in triage acuity may reflect true changes in patient severity, instrument revisions, or shifts in clinicians education level or behavior that cannot be disentangled. An important limitation is that non-transported patients were not included in the outcome analyses, despite the observed increase in this group, since it was not within the aim of this study. We lacked data on diagnoses, comorbidities, and socioeconomic status, which would have allowed more granular characterization of the patient population.

## Conclusions

The sustained upward shift in acuity, combined with the expanding role of EMS in on-scene disposition, suggests that future EMS organizations should anticipate continued growth in clinical decision-making complexity. The increasing share of hospital admissions attributable to EMS-transported patients also highlights the need to integrate prehospital data into hospital capacity planning. Prospective studies examining outcomes of non-transported patients, the impact of triage system revisions on classification accuracy, and the interaction between EMS acuity trends and hospital bed availability would advance understanding of the dynamics identified in this study.

## Supplementary Information

Below is the link to the electronic supplementary material.

Supplementary Material 1

## Data Availability

An anonymized dataset used and analysed during the current study is available on reasonable request to the authors.

## Abbreviations

CI: Confidence Interval
ED: Emergency Department
HER: Electronic Health Records
EMS: Emergency Medical Services
NEMSIS: National Emergency Medical Information System
OHCA: Out of Hospital Cardiac Arrest
OR: Odds Ratio
RETTS: Rapid Emergency Triage and Treatment System
