# Supplementary material for: The evolving prehospital care: a 12-year retrospective analysis
Source: Scand J Trauma Resusc Emerg Med. 2026 Jul 1;34:115. doi: 10.1186/s13049-026-01651-z (PMC13330154; doi:10.1186/s13049-026-01651-z)
Supplement: Supplementary file 1 — Supplementary Material 1 [file 13049_2026_1651_MOESM1_ESM.pdf]

Additional file 1: Summary table of the data showing total number of all variables (and proportion or SD) between years 2011 to 2022.

	**2011**	**2012**	**2013**	**2014**	**2015**	**2016**	**2017**	**2018**	**2019**	**2020**	**2021**	**2022**
All EMS assignments	51743	57031	57726	56638	55467	47701	48705	49200	51214	52598	56909	55007
Primary assignment total	42659	47523	48554	47547	47840	47238	45283	45372	46728	47620	51085	49521
Referral/Treatment on scene	2219	3001	3394	3606	4165	4960	5920	7798	8648	11475	12557	12351
**Primary assignment to ED** N =	**31043**	**33675**	**34733**	**34148**	**33955**	**33196**	**33477**	**32392**	**32450**	**30604**	**32467**	**32440**
Women (N (%))	16085 (51.8)	17512 (52.0)	18056 (52.0)	17898 (52.4)	17741 (52.2)	17337 (52.2)	17319 (51.7)	16603 (51.3)	16694 (51.4)	15666 (51.2)	16733 (51.5)	16707 (51.5)
Men (N (%))	14958 (48.2)	16163 (48.0)	16677 (48.0)	16250 (47.6)	16214 (47.8)	15859 (47.8)	16158 (48.3)	15789 (48.7)	15756 (48.6)	14938 (48.8)	15734 (48.5)	15733 (48.5)
Age (mean (SD))	64.1 (22.7)	64.0 (22.7)	64.0 (22.9)	63.8 (23.4)	64.5 (23.2)	63.6 (23.9)	64.1 (23.7)	65.0 (23.3)	64.9 (23.5)	65.5 (22.8)	65.7 (23.0)	66.2 (22.9)
Day (06-17) N (%)	17345 (55.9)	19001 (56.4)	19505 (56.1)	19206 (56.2)	19422 (57.2)	18842 (56.8)	19108 (57.1)	18712 (57.8)	18347 (56.5)	17433 (57.0)	18256 (56.2)	18376 (56.6)
Evening (17-22) N (%)	7096 (22.8)	7599 (22.6)	7769 (22.4)	7641 (22.4)	7589 (22.3)	7444 (22.4)	7400 (22.1)	7249 (22.4)	7285 (22.5)	6958 (22.7)	7427 (22.9)	7394 (22.8)

Night (22-06) N (%)	6602 (21.3)	7075 (21.0)	7459 (21.5)	7301 (21.4)	6944 (20.5)	6910 (20.8)	6969 (20.8)	6431 (19.8)	6818 (21.0)	6213 (20.3)	6784 (20.9)	6670 (20.6)
ED Urban tertiary care center (N (%))	11643 (37.5)	12760 (37.9)	13063 (37.6)	12779 (37.4)	12822 (37.8)	13047 (39.3)	12738 (38.0)	12403 (38.3)	12498 (38.5)	11332 (37.0)	11447 (35.3)	11669 (36.0)
ED Urban community hospital (N (%))	13545 (43.6)	14649 (43.5)	15211 (43.8)	15120 (44.3)	15002 (44.2)	14318 (43.1)	14525 (43.4)	13700 (42.3)	13902 (42.9)	13780 (45.0)	15170 (46.7)	15131 (46.6)
ED Rural community hospital (N (%))	5855 (18.9)	6266 (18.6)	6459 (18.6)	6249 (18.3)	6131 (18.0)	5831 (17.6)	6214 (18.6)	6289 (19.4)	6050 (18.6)	5492 (18.0)	5850 (18.0)	5640 (17.4)
Admissions (all EDs) (N (%))	14360 (46.3)	17008 (50.5)	17247 (49.7)	16945 (49.6)	16235 (47.8)	15727 (47.4)	15587 (46.6)	15622 (48.2)	15510 (47.8)	16564 (54.1)	17748 (54.7)	17606 (54.3)
Acuity level 1 (red)	2613 (8.4)	3030 (9.0)	3565 (10.3)	3526 (10.3)	3445 (10.2)	3113 (9.4)	3255 (9.7)	3159 (9.8)	3005 (9.3)	3065 (10.0)	3287 (10.1)	3812 (11.8)
Acuity level 2 (orange)	7330 (23.6)	8756 (26.0)	10358 (29.8)	10582 (31.0)	11210 (33.0)	13218 (39.9)	13542 (40.5)	13361 (41.2)	13574 (41.8)	13336 (43.6)	14454 (44.5)	14791 (45.6)
Acuity level 3 (yellow)	14855 (47.9)	15917 (47.3)	15847 (45.6)	15798 (46.3)	15388 (45.3)	13288 (40.0)	13010 (38.9)	12937 (39.9)	13096 (40.4)	11789 (38.5)	12274 (37.8)	11693 (36.0)
Acuity level 4 (green)	5995 (19.3)	5772 (17.1)	4801 (13.8)	4114 (12.0)	3814 (11.2)	3496 (10.5)	3559 (10.6)	2837 (8.8)	2665 (8.2)	2316 (7.6)	2338 (7.2)	2058 (6.3)

Missing acuity level	250 (0.8)	200 (0.6)	162 (0.5)	128 (0.4)	98 (0.3)	81 (0.2)	111 (0.3)	98 (0.3)	110 (0.3)	98 (0.3)	114 (0.4)	86 (0.3)
Mortality within 1 day (N (%))	304 (1.0)	319 (0.9)	315 (0.9)	338 (1.0)	356 (1.0)	374 (1.1)	341 (1.0)	401 (1.2)	331 (1.0)	355 (1.2)	375 (1.2)	369 (1.1)
Mortality within 7 days (N (%))	393 (1.3)	463 (1.4)	451 (1.3)	459 (1.3)	455 (1.3)	438 (1.3)	539 (1.6)	472 (1.5)	462 (1.4)	515 (1.7)	557 (1.7)	468 (1.4)
Mortality within 30 days (N (%))	732 (2.4)	825 (2.4)	816 (2.3)	834 (2.4)	904 (2.7)	816 (2.5)	835 (2.5)	869 (2.7)	856 (2.6)	960 (3.1)	983 (3.0)	920 (2.8)
Mortality within 90 days (N (%))	1007 (3.2)	1128 (3.3)	1001 (2.9)	1117 (3.3)	1135 (3.3)	1112 (3.3)	1079 (3.2)	1046 (3.2)	1146 (3.5)	1223 (4.0)	1209 (3.7)	1234 (3.8)
